# Neutralizing Antibody Response to *Sarbecovirus* Is Delayed in Sequential Heterologous Immunization

**DOI:** 10.3390/v14071382

**Published:** 2022-06-24

**Authors:** Huibin Lv, Ray T. Y. So, Qi Wen Teo, Meng Yuan, Hejun Liu, Chang-Chun D. Lee, Garrick K. Yip, Wilson W. Ng, Ian A. Wilson, Malik Peiris, Nicholas C. Wu, Chris Ka Pun Mok

**Affiliations:** 1 HKU-Pasteur Research Pole, School of Public Health, Li Ka Shing Faculty of Medicine, The University of Hong Kong, Hong Kong SAR, China; huibin01@connect.hku.hk (H.L.); soty87@connect.hku.hk (R.T.Y.S.); qiwenteo@illinois.edu (Q.W.T.); garrick.yip@c2i.hk (G.K.Y.); wilson.ng@c2i.hk (W.W.N.); malik@hku.hk (M.P.); 2 Carl R. Woese Institute for Genomic Biology, University of Illinois at Urbana-Champaign, Urbana, IL 61801, USA; 3 Department of Integrative Structural and Computational Biology, The Scripps Research Institute, La Jolla, CA 92037, USA; myuan@scripps.edu (M.Y.); heliu@scripps.edu (H.L.); chlee@scripps.edu (C.-C.D.L.); wilson@scripps.edu (I.A.W.); 4 The Skaggs Institute for Chemical Biology, the Scripps Research Institute, La Jolla, CA 92037, USA; 5 Department of Biochemistry, University of Illinois at Urbana-Champaign, Urbana, IL 61801, USA; 6 Center for Biophysics and Computational Biology, University of Illinois at Urbana-Champaign, Urbana, IL 61801, USA; 7 Carle Illinois College of Medicine, University of Illinois at Urbana-Champaign, Urbana, IL 61801, USA; 8 The Jockey Club School of Public Health and Primary Care, The Chinese University of Hong Kong, Hong Kong SAR, China; 9 Li Ka Shing Institute of Health Sciences, Faculty of Medicine, The Chinese University of Hong Kong, Hong Kong SAR, China

**Keywords:** SARS-CoV-2, antigenic imprinting, coronavirus, COVID-19

## Abstract

Antigenic imprinting, which describes the bias of the antibody response due to previous immune history, can influence vaccine effectiveness. While this phenomenon has been reported for viruses such as influenza, there is little understanding of how prior immune history affects the antibody response to SARS-CoV-2. This study provides evidence for antigenic imprinting through immunization with two *Sarbecoviruses*, the subgenus that includes SARS-CoV-2. Mice were immunized subsequently with two antigenically distinct *Sarbecovirus* strains, namely SARS-CoV-1 and SARS-CoV-2. We found that sequential heterologous immunization induced cross-reactive binding antibodies for both viruses and delayed the emergence of neutralizing antibody responses against the booster strain. Our results provide fundamental knowledge about the immune response to *Sarbecovirus* and important insights into the development of pan-sarbecovirus vaccines and guiding therapeutic interventions.

## 1. Introduction

Antigenic imprinting describes the preferential reactivation of antibodies against cross-reactive epitopes following an infection or immunization with antigenically related, but non-identical viruses [1,2]. In many cases, these antibodies are not able to neutralize the second virus involved in a sequential infection or immunization and are considered to hamper protective responses. The effect of antigenic imprinting has been found in many different virus families including the influenza virus [3,4], dengue virus [5], and HIV [6]. It was observed during the 2009 swine-origin influenza pandemic (H1N1) outbreak, where exposure to the pandemic H1N1 virus preferentially induced antibodies from the memory B cells of previous seasonal influenza infections [7,8]. In fact, it is now recognized that the effectiveness of a given influenza vaccine varies among people with different influenza immunization or infection histories, this may be influenced by antigenic imprinting elicited by the first-infecting virus [9,10,11].

The Coronavirus 2019 (COVID-19) pandemic is an ongoing global public health crisis and has imposed a huge burden on economic and social well-being. The causative virus of COVID-19, SARS-CoV-2, belongs to the subgenus *Sarbecovirus* of the genus *Betacoronavirus* and has nearly 80% genomic sequence identity with another *Sarbecovirus*, SARS-CoV-1, which caused a human epidemic in 2003 [12]. Other human seasonal coronaviruses, which belong to the betacoronavirus group such as HCoV-OC43 or HCoV-HKU1, also share a moderate level of similarity with SARS-CoV-2. Although many SARS-CoV-2 vaccine candidates have been developed [13,14], the influence of previous immune history (infection or vaccination) on vaccine effectiveness remains largely uncharacterized. Our previous study showed an induction of cross-reactive binding antibodies to the spike proteins of SARS-CoV-1 and SARS-CoV-2 during either infection or immunization, suggesting the potential for antigenic imprinting [15]. Since SARS-CoV-2 is likely to become a seasonal human coronavirus in future [16] and other zoonotic *Sarbecovirus* strains continue to pose a pandemic threat [17], it is likely that antigenically diverse variants of SARS-CoV-2 will arise in future. There is an initiative to develop broadly protective pan-coronavirus vaccines [18], and it is important, therefore, to understand how antigenic imprinting may affect our antibody responses to *Sarbecovirus*. In this study, we explored how antigenic imprinting influences the antibody response to *Sarbecovirus* by sequential immunization of mice with SARS-CoV-1 and SARS-CoV-2.

## 2. Materials and Methods

### 2.1. Virus Culture

Patient-derived SARS-CoV-1 (strain HK39849, SCoV) and SARS-CoV-2 (BetaCoV/Hong Kong/VM20001061/2020 [KH1]) were isolated from The University of Hong Kong and passaged in Vero-E6 cells (ATCC CRL-1586). The virus stock was aliquoted and titrated by determining a tissue culture infection dose of 50% (TCID_50_) in Vero-E6 cells.

### 2.2. RBD Protein Expression and Purification

The receptor-binding domain (RBD) (residues 319–541) of the SARS-CoV-2 spike (S) protein (GenBank: QHD43416.1) and RBD (residues: 306–527) of the SARS-CoV-1 spike (S) protein (GenBank: ABF65836.1) were cloned into a customized pFastBac vector [19] and fused with an N-terminal gp67 signal peptide and C-terminal 6 × His-tag [20]. A recombinant bacmid DNA was generated using the Bac-to-Bac system (Thermo Fisher Scientific, Waltham, MA, USA). The baculovirus was generated by transfecting purified bacmid DNA into Sf9 cells using FuGENE HD (Promega, Madison, WI, USA), and subsequently used to infect suspension cultures of High Five cells (Thermo Fisher Scientific, Waltham, MA, USA) at an MOI of 5 to 10. For protein expression, the infected High Five cells were incubated at 28 °C for 72 h with shaking at 110 r.p.m. The supernatant was then concentrated using a 10 kDa MW cutoff Centramate cassette (Pall Corporation, Washington, DC, USA). The RBD protein was purified by Ni-NTA, followed by size exclusion chromatography, and buffer exchanged into 20 mM Tris-HCl pH 7.4 and 150 mM NaCl.

### 2.3. ACE2 Protein Expression and Purification

The expression of human ACE2 was as previously reported [21]. Briefly, the human ACE2 (residues 19 to 615, GenBank: BAB40370.1) was codon optimized and cloned into the phCMV3 vector [21]. The construct was fused with a C-terminal 6xHis tag. The plasmid was transiently transfected into Expi293F cells using ExpiFectamine 293 Reagent (Thermo Fisher Scientific, Waltham, MA, USA) according to the manufacturer’s manual. At 6 days post-transfection, the supernatant was harvested and was then washed and eluted with 10 mM and 300 mM Imidazole containing PBS, respectively. The ACE2 eluent was purified by size exclusion chromatography.

### 2.4. Mouse Immunization

First, 6–8 week BALB/c mice were immunized with two rounds 10^5^ pfu of viruses in 150 μL PBS mixing with 50 μL Addavax as previously described [22]. Our immunization schemes include: (1) two rounds of homologous SARS-CoV-1 immunization, (2) two rounds of heterologous immunization with SARS-CoV-1-prime and SARS-CoV-2-boost, (3) two rounds of homologous SARS-CoV-2 immunization, and (4) two rounds of heterologous immunization with SARS-CoV-2-prime and SARS-CoV-1-boost, via intraperitoneal (i.p.) route. The blood samples were collected using heparin tubes on day 14 or day 21 after the second round of immunization. Plasma samples were separated by centrifugation and PBMCs were isolated by Ficoll-Paque according to the manufacturer’s protocol (GE Healthcare, Chicago, IL, USA). The experiments were conducted in the University of Hong Kong Biosafety Level 3 (BSL3) facility. This study protocol was carried out in strict accordance with the recommendations and was approved by the Committee on the Use of Live Animals in Teaching and Research of the University of Hong Kong (CULATR 5422-20, Date of approval: 29 May 2020).

### 2.5. ELISA Binding Assay

ELISA were performed by serial dilution of the immunized mouse plasma samples. Binding values from the dilution curves were used to determine the area under the curve (AUC). ELISA plates (96-well, Nunc MaxiSorp, Thermo Fisher Scientific, Waltham, MA, USA) were coated overnight with 100 μL of purified recombinant protein in PBS buffer at 1 ng/μL. The plates were then blocked with 100 μL Chonblock buffer (Chondrex Inc., Redmon, IL, USA) at room temperature for 1 h. Each mouse plasma sample was diluted in Chonblock buffer, added to the coated ELISA plates, and incubated for 2 h at 37 °C. After three extensive washes with PBS containing 0.1% Tween 20, each well was incubated with the HRP goat anti-mouse secondary antibody (1:5000, Beyotime Biotechnology, Shanghai, China) for 1 h at 37 °C. The ELISA plates were then washed five times with PBS containing 0.1% Tween 20. Subsequently, 100 μL of TMB buffer (Ncm TMB One; New Cell & Molecular Biotech Co., Ltd., Suzhou, China) was added into each well. After 15 min incubation, 50 μL of 2 M H_2_SO_4_ solution was added to stop the reaction and the plates were analyzed on a Sunrise absorbance microplate reader (Tecan, Männedorf, Switzerland) at 450 nm wavelength.

### 2.6. ACE2-Competition ELISA Assay

The ELISA plates were coated overnight with 100 ng of SARS-CoV-1 or SARS-CoV-2 RBD protein in PBS buffer. The plates were then blocked with 100 μL Chonblock buffer (Chondrex Inc., Redmon, IL, USA) at room temperature for 1 h. After washing, 100 ng of ACE2 protein was added into plate and incubated at 37 °C for 2 h, followed by another 2 h of 1:200 diluted mouse plasma samples incubation. After three extensive washes with PBS containing 0.1% Tween 20, each well was incubated with the HRP goat anti-mouse secondary antibody (1:5000, Beyotime Biotechnology, Shanghai, China) for 1 h at 37 °C. The ELISA plates were then washed five times with PBS containing 0.1% Tween 20. Subsequently, 100 μL of TMB buffer was added into each well. After 15 min incubation, 50 μL of 2 M H_2_SO_4_ solution was added to stop the reaction and the plates were analyzed on a Sunrise absorbance microplate reader at 450 nm wavelength.

### 2.7. Plaque Reduction Neutralization Test (PRNT)

Plasma samples were two-fold diluted, starting from a 1:10 dilution, and mixed with equal volumes of 120 plaque-forming units (pfu) of SARS-CoV-1 or SARS-CoV-2 as determined by Vero E6 cells. After 1 h incubation at 37 °C, the plasma-virus mixture was added onto Vero E6 monolayers seated in a 24-well cell culture plate and incubated for 1 h at 37 °C with 5% CO_2_. The plasma–virus mixtures were then discarded and infected Vero E6 cells were immediately covered with 1% agarose gel in DMEM medium. After incubation for 3 days at 37 °C with 5% CO_2_, the plates were formalin fixed and stained by 0.5% crystal violet solution. Neutralization titers were determined by the highest plasma dilution that resulted in >90% reduction in the number of pfus. The test was performed in a BSL3 facility in the University of Hong Kong.

### 2.8. Enrichment of SARS-CoV-1 and SARS-CoV-2 RBD Binding B Cells

PBMCs isolated 14 days after two rounds of immunization from five mice of the same group were pooled together. The cells were first stained with 10 μL of anti-mouse CD19-APC (BioLegend, San Diego, CA, USA), 2 μg of SARS-CoV-1 RBD-BB515 and 2 μg SARS-CoV-2 RBD-PE and incubated for 1 h at 4 °C. Cells were washed by PBS and then fixed with 4% PFA for 20 min. The percentage of B cells that showed double positive of BB515 and PE were determined via flow cytometry (AttuneNxT) and analyzed by FlowJo v10. 

## 3. Results

### 3.1. Antigenic Imprinting in Mice after Heterologous Immunization of Sarbecovirus

To investigate if antigenic imprinting might occur after infection by *Sarbecovirus*, we immunized mice with homologous or heterologous combinations of SARS-CoV-1 and SARS-CoV-2 viruses using different prime-boost strategies and determined the serological response against these two viruses. The mice were divided into four groups and immunized by one of the following strategies: (Group 1) two sequential doses of SARS-CoV-1 (SARS-CoV-1 homologous prime-boost); (Group 2) first dose with SARS-CoV-1 and second dose with SARS-CoV-2 (heterologous SARS-CoV-1-prime, SARS-CoV-2-boost); (Group 3) two doses of SARS-CoV-2 (SARS-CoV-2 homologous prime-boost); and (Group 4) first dose of SARS-CoV-2 and second dose of SARS-CoV-1 (heterologous SARS-CoV-2-prime, SARS-CoV-1-boost). Antibody levels against the two subtypes were measured in plasma samples collected on day 14 after the second round of immunization. To determine the immunogenicity of the two viruses in a naïve background, we first measured the antibody levels from the mice that were immunized only once with either SARS-CoV-1 or SARS-CoV-2. As expected, both viruses could stimulate the induction of binding and neutralizing antibodies against their corresponding immunized strain 14 days after one dose of immunization, as measured by ELISA and PRNT_90,_ respectively (Figure S1). Two immunizations with a homologous or heterologous prime-boost strategy induced significantly higher binding antibody titers than one round of immunization (*p* < 0.05; Figure S1A–D). These results suggest that both SARS-CoV-1 and SARS-CoV-2 are immunogenic in our mouse model.

**Figure 1:**
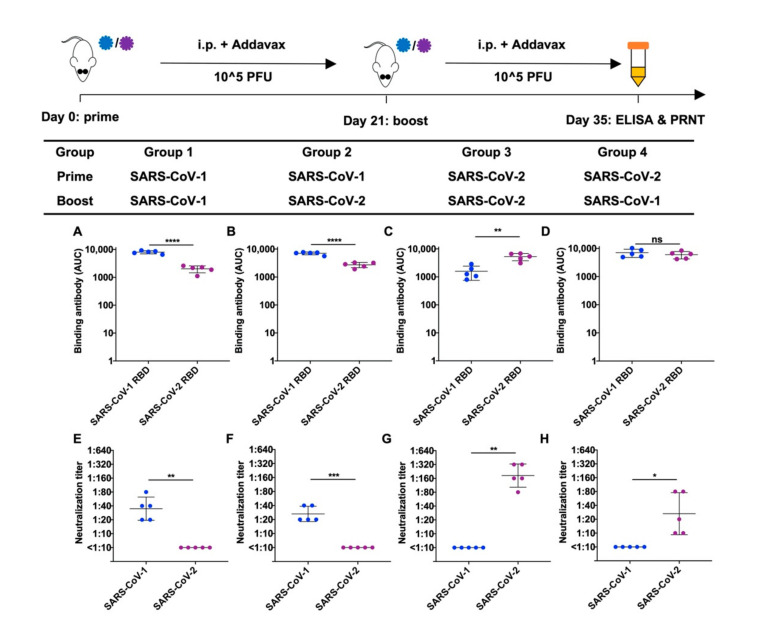
Binding and neutralization of mouse plasma 14 days after homologous or heterologous immunization of SARS-CoV-1 and SARS-CoV-2. Plasma samples were collected 14 days (day 35) after two rounds of immunization (prime-day 0, boost-day 21) with SARS-CoV-1 or SARS-CoV-2 as illustrated. (**A**–**D**) RBD (receptor binding domain) proteins from SARS-CoV-1 and SARS-CoV-2 were used as the antigen coating on the ELISA plates. Binding of RBD to serial diluted plasma sample was analyzed from five mice immunized using (**A**) SARS-CoV-1 homologous prime-boost, (**B**) heterologous SARS-CoV-1-prime, SARS-CoV-2-boost, (**C**) SARS-CoV-2 homologous prime-boost, and (**D**) heterologous SARS-CoV-2-prime, SARS-CoV-1-boost. AUC, area under the curve. (**E**–**H**) Neutralizing titers (PRNT_90_) of plasma samples from mice immunized with (**E**) SARS-CoV-1 homologous prime-boost, (**F**) heterologous SARS-CoV-1-prime, SARS-CoV-2-boost, (**G**) SARS-CoV-2 homologous prime-boost, and (**H**) heterologous SARS-CoV-2-prime, SARS-CoV-1-boost, were analyzed by a PRNT (plaque reduction neutralization test) assay. Each data point in the figure represents the mean of two replicates. Error bars represent standard deviation. *p*-values were calculated using a two-tailed *t*-test (* *p* < 0.05, ** *p* < 0.01, *** *p* < 0.001, **** *p* < 0.0001, ns (not significant)).

Next, we compared the binding and neutralizing antibody levels in mice immunized with different prime-boost strategies (Figure 1). Both homologous and heterologous immunization resulted in the induction of binding antibody against the RBDs of both SARS-CoV-1 and SARS-CoV-2 (Figure 1A–D). However, we found that the plasma samples collected from the mice with the heterologous immunization only neutralized the virus that was used for the priming immunization (Figure 1E–H). This finding suggests that prior immune exposure may influence the antibody response against an antigenically similar strain.

To further confirm whether cross-reactive antibodies to the two SARS-CoVs in the mice were induced after heterologous immunization, PBMCs isolated from the mice of the same immunization group (*n* = 5) collected on day 14 after two rounds of immunization were pooled together and the B cells that showed binding to both the RBDs of SARS-CoV-1 and SARS-CoV-2 were identified (Figure S2). Our results showed that either homologous or heterologous immunization could stimulate the induction of cross-reactive B cells that target to the RBDs of both SARS-CoV-1 and SARS-CoV-2.

### 3.2. Delayed Neutralizing Response in Heterologous Immunization

**Figure 2:**
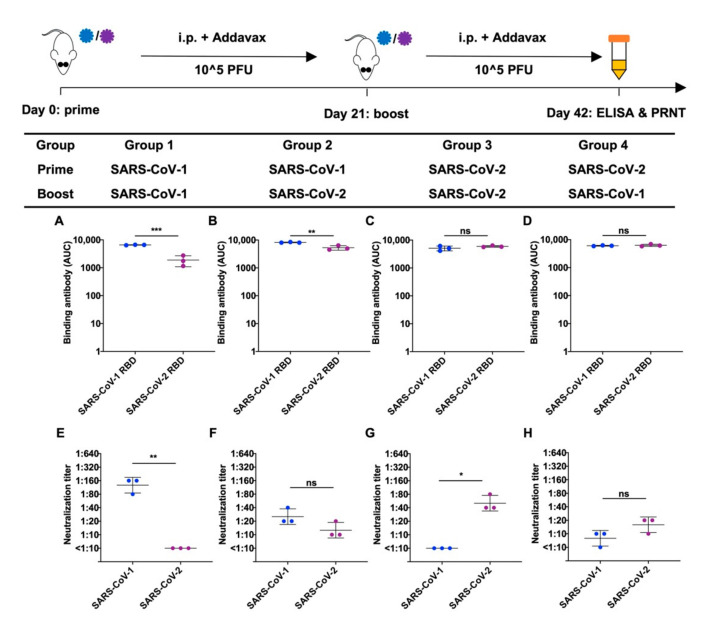
Binding and neutralization of antibodies in mouse plasma 21 days after the homologous or heterologous immunization of SARS-CoV-1 and SARS-CoV-2. Plasma samples were collected on day 21 after two rounds of immunization. (**A**–**D**) RBD (receptor binding domain) proteins from SARS-CoV-1 and SARS-CoV-2 were used as the antigen coating on the ELISA plates. Binding of RBD to serial diluted plasma sample was analyzed from three mice immunized using (**A**) SARS-CoV-1 homologous prime-boost, (**B**) heterologous SARS-CoV-1-prime, SARS-CoV-2-boost, (**C**) SARS-CoV-2 homologous prime-boost, and (**D**) heterologous SARS-CoV-2-prime, SARS-CoV-1-boost. AUC, area under the curve. (**E**–**H**) Neutralizing titers (PRNT_90_) of plasma samples from mice immunized with (**E**) SARS-CoV-1 homologous prime-boost, (**F**) heterologous SARS-CoV-1-prime, SARS-CoV-2-boost, (**G**) SARS-CoV-2 homologous prime-boost, and (**H**) heterologous SARS-CoV-2-prime, SARS-CoV-1-boost, were analyzed by a PRNT (plaque reduction neutralization test) assay. Each data point in the figure represents the mean of two replicates. Error bars represent standard deviation. *p*-values were calculated using a two-tailed *t*-test (* *p* < 0.05, ** *p* < 0.01, *** *p* < 0.001, ns (not significant)).

Although we observed an absence of neutralizing antibodies against the virus used for the second dose in the heterologous immunization schedule, it was reported that neutralizing antibodies to SARS-CoV-2 were detected in SARS-CoV-1 convalescent patients after they received the BNT162b2 mRNA vaccine [23]. We thus suspected if the time between the two rounds of immunization might affect the outcome. We repeated our experiments with the same immunization strategies but collected the plasma samples from the mice on day 21 after they received the second vaccine dose. Similar to the results from plasma collected on day 14 (Figure 1A–D), SARS-CoV-1 and SARS-CoV-2 RBD binding antibodies could be detected in all four prime-boost immunization strategies (Figure 2A–D). Interestingly, the mice with heterologous immunization showed neutralizing titers–albeit weak–against the virus used for the second vaccine dose (Figure 2E–H). To address if the higher binding titer to the booster virus contributed to the presence of neutralizing titer at day 21, we compared the results of the binding titers between day 14 and day 21 in group 2 (SARS-CoV-1 then SARS-CoV-2) and group 4 (SARS-CoV-2 then SARS-CoV-1). In group 2, the binding to SARS-CoV-2 in day 21 was significantly higher than that of day 14 (*p* = 0.0011). However, no significant difference was found in group 4 (*p* = 0.92). Since both group 2 and 4 showed the presence of neutralizing antibody to the booster virus at day 21 in the plasma, our results did not support that higher neutralizing activity was contributed by the higher antigen binding titer.

**Figure 3:**
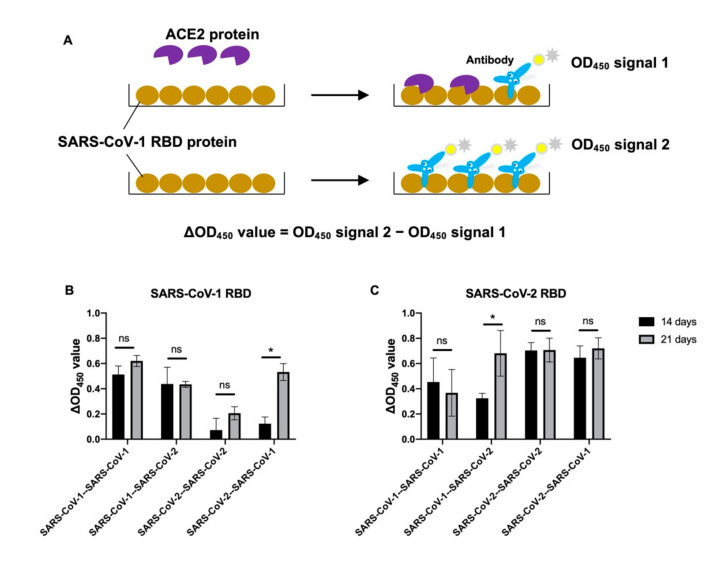
Competition of ACE2 for binding of antibodies in mouse plasma from homologous or heterologous immunization of SARS-CoV-1 and SARS-CoV-2 (**A**) ACE2 competition schematic. SARS-CoV-1 or SARS-CoV-2 RBD protein was captured on the plate. ACE2 protein was premixed with RBD on the plate to test as competitor to block antibody binding to the RBD. The OD_450_ value is determined from an anti-human-HRP colorimetric readout. Plasma samples were collected on day 14 or 21 after two rounds of immunization. (**B**) SARS-CoV-1 RBD or (**C**) SARS-CoV-2 RBD protein was used as antigen for ELISA. The ΔOD_450_ values were calculated from the difference between the results of the same plasma with and without hACE2 protein as a blocking agent. Each condition is represented by the mean of the results obtained from at least three mice. *p*-values were calculated using a two-tailed *t*-test (* *p* < 0.05, ns (not significant)).

A previous study showed that the neutralization titers in the blood are correlated with the level of antibodies that can block the interaction between SARS-CoV-2 RBD and angiotensin-converting enzyme 2 (ACE2) [24]. Since we found that heterologous immunization induced substantial level of binding antibodies against the RBD of both subtypes, we thus established an ACE2-competition assay to determine the level of antibody in the plasma that binds to the same epitopes as RBD, which are used for interacting with ACE2 protein (Figure 3). ACE2 was first incubated with the RBD so that it could occupy the interacting epitopes. The antibodies in the plasma thus can only bind to the regions that do not participate in the RBD–ACE2 interaction. In this assay, plasma samples with stronger ACE2 competition activity will show higher reduction in antibody binding signal to RBD when the ACE2 protein is added as a competitive agent in the ELISA assay (Figure 3A). We used ΔOD_450_ value to represent the level of antibody that binds to the ACE2 binding epitope. Of note, a high ΔOD_450_ value means increased ACE2 competition from day 14 to day 21, while a low ΔOD_450_ value indicates there is less or change between day 14 and day 21. Interestingly, there was a significant increase in ACE2 competition activity on SARS-CoV-1 RBD in the plasma of the mice collected at 21 days after heterologous SARS-CoV-2/SARS-CoV-1 immunization. This result is consistent with the observation that neutralization against SARS-CoV-1 was found at 21 days but not at 14 days after heterologous immunization using this strategy (Figure 1H and Figure 2H). Similar results were also found in SARS-CoV-2 RBD ELISA in the plasma samples collected at 21 days from the mice with SARS-CoV-1/SARS-CoV-2, as the prime/boost subtypes showed significantly higher ACE2 competition activity than those collected at day 14 under the same conditions (Figure 3B, C).

## 4. Discussion

In this study, we compared the antibody responses in different prime-boost immunization strategies and we found that the second dose could presumably induce cross-reactive binding antibodies against conserved regions in SARS-CoV-1 and SARS-CoV-2 during our heterologous immunization. However, the induction of neutralizing antibodies against the booster virus was found to be delayed compared to the single round immunization by the same virus. The results using the same mouse model from our previous study showed that the level of cross-reactive binding antibody between SARS-CoV-1 and SARS-CoV-2 was low if the mice were only immunized once with either one of the viruses [15]. While the homologous immunization clearly induced cross-reactive antibody between SARS-CoV-1 and SARS-CoV-2 in the mice, the antibodies induced by the heterologous immunizations need to be further characterized. Importantly, our results from flow cytometry (Figure S2) further determined that the cross-reactive B cells were present on day 14 in the mice that had received homologous or heterologous immunization. The findings suggest that the B cells that were induced at 14 days after heterologous immunization still only targeted to the non-neutralizing epitopes. The booster strain specific neutralizing antibody would be then subsequently generated through new rounds of somatic hypermutation during the next seven days. In conclusion, our results demonstrate that antigenic imprinting may have occurred between the distantly related *Sarbecoviruses*, SARS-CoV-1 and SARS-CoV-2.

A recent study has indeed indicated that pre-existing antibodies against human seasonal coronaviruses may impede the generation of the SARS-CoV-2 neutralizing antibodies in the mouse model [25]. In addition, such pre-existing antibodies could not provide a SARS-CoV-2 neutralizing function [26] and may negatively correlate with the induction of SARS-CoV-2 spike specific antibodies [27] so as to increase susceptibility to SARS-CoV-2 [28]. It is interesting to note that a similar phenomenon was also observed when convalescent patients of Dengue virus (DENV) infection were subsequently infected by the Zika virus (ZIKV) [29]. Specifically, a decreased Zika-specific memory B cell population was noticed in DENV-experienced, ZIKV-infected individuals. This may be contributed by the reactivation of memory B cells that target cross-reactive regions during the early stage of ZIKV infections [30,31,32]. Altogether, the delay of specific antibody responses at early times of infection could reflect the propensity to activate pre-existing and cross-reactive B cells against an antigenically related virus and may impact on the pathogenesis of an infection.

Understanding the dynamics of antibody responses to SARS-CoV-2 is critical for optimizing clinical management regimens against COVID-19. A recent study showed that the development of neutralizing antibodies during the early stage of infection correlates with the time to viral clearance and the mortality rate [33]. This observation indicates that early development of neutralizing antibodies against SARS-CoV-2 is critical for patient survival and virus control. Thus, prior immune history that may affect the early induction of a neutralizing response to SARS-CoV-2 can have important implications in prognosis and in the guidance of treatment options, including passive immunotherapy. Similarly, the design of next-generation vaccines against COVID-19 may need to focus on minimizing the negative impact of antigenic imprinting, however, further investigation will be needed to understand the role of the antigenic imprinting during infection. While several promising candidates of broadly protective SARS-CoV-2 vaccines have been developed [34,35], it will be important to assess whether their continued effectiveness is influenced by prior immune history.

## Data Availability

Not applicable.

## Supplementary Materials

The following supporting information can be downloaded at: https://www.mdpi.com/article/10.3390/v14071382/s1, Figure S1: Binding and neutralizing antibodies after one or two rounds of homologous SARS-CoV-1 or SARS-CoV-2 immunization; Figure S2. Gating strategy for SARS-CoV-1 and SARS-CoV-2 RBD-specific B cells.
