# Supplementary material for: Neutralizing Antibody Response to Sarbecovirus Is Delayed in Sequential Heterologous Immunization
Source: Viruses. 2022 Jun 24;14(7):1382. doi: 10.3390/v14071382 (PMC9318566; doi:10.3390/v14071382)
Supplement: Supplementary file 1 [file viruses-14-01382-s001.zip › viruses-1766442-Supplementary Figures.pdf]

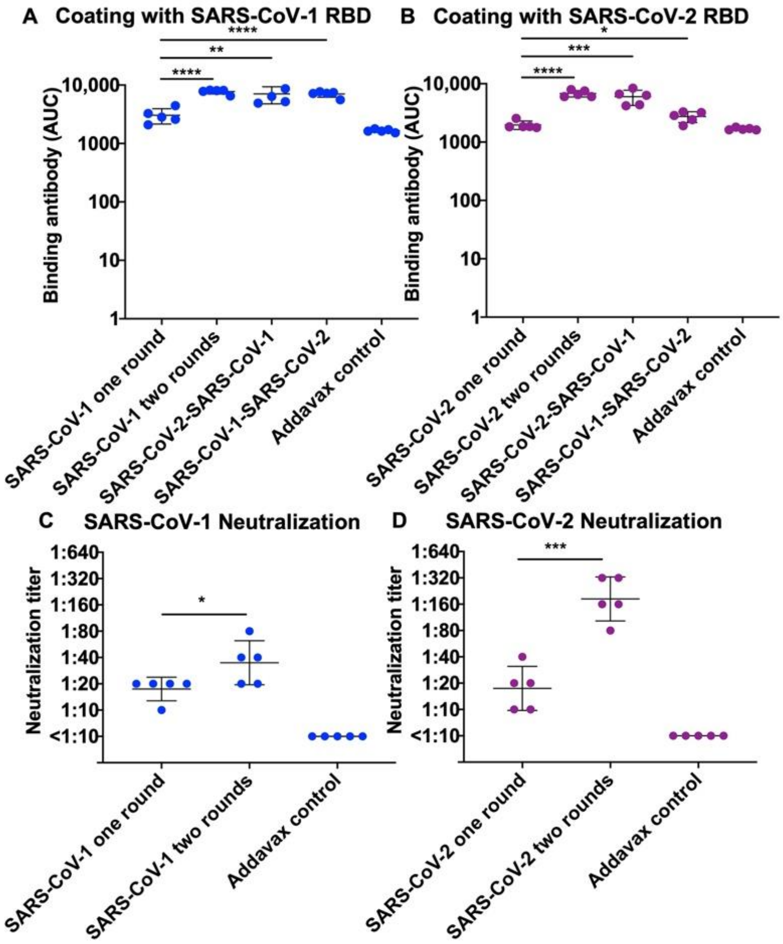

Figure S1. Binding and neutralizing antibodies after one or two rounds of homologous SARS-CoV-1 or SARS-CoV-2 immunization. **(A-B)** Binding antibody levels in plasma samples from mice immunized by one or two rounds of homologous or heterologous virus after day 14 against **(A)** SARS-CoV-1 RBD or **(B)** SARS-CoV-2 RBD were measured by ELISA assay. AUC, area under the curve. **(C-D)** Neutralizing titers of plasma samples from mice immunized by one or two rounds of homologous virus after day 14 against **(C)** SARS-CoV-1 or **(D)** SARS-CoV-2 were measured by a PRNT assay. Each data point in the figure represents the mean of two technical replicates. *p*-values were calculated using two-tailed t-test (* $p < 0.05$, ** $p < 0.01$, *** $p < 0.001$, **** $p < 0.0001$). Error bars represent standard deviation.

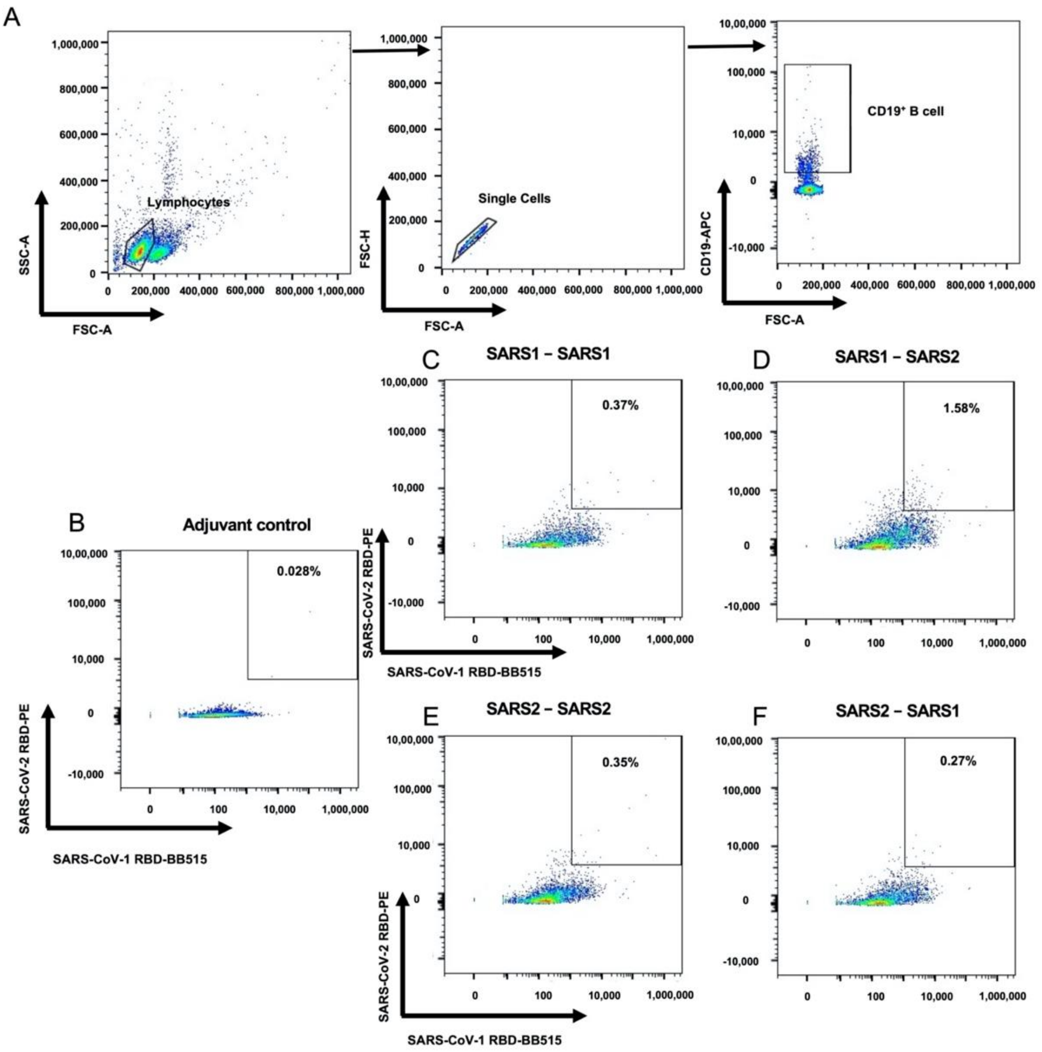

Figure S2. Gating strategy for SARS-CoV-1 and SARS-CoV-2 RBD-specific B cells. PBMCs isolated from the mice of the same immunization group (n=5) collected on day 14 after two rounds of immunization were pooled together and the cells were stained with anti-mouse CD19-APC, SARS-CoV-1-RBD-BB515 and SARS-CoV-2-RBD-PE. The data was acquired by flow cytometry and the B cells were identified by the gating strategy in (A). Percentages indicate the proportions of $CD19^+$SARS-CoV-1 RBD^+ SARS-CoV-2 RBD^+ B cells among all B cells in different experimental conditions (B) Adjuvant only (C) SARS-CoV-1 homologous prime-boost (D) SARS-CoV-1-prime, SARS-CoV-2-boost, (E) SARS-CoV-2 homologous prime-boost (F) SARS-CoV-2-prime, SARS-CoV-2-boost.
